# Translational recoding by chemical modification of non-AUG start codon ribonucleotide bases

**DOI:** 10.1126/sciadv.abm8501

**Published:** 2022-04-08

**Authors:** Yoshihiko Fujita, Takeru Kameda, Chingakham Ranjit Singh, Whitney Pepper, Ariana Cecil, Madelyn Hilgers, Mackenzie Thornton, Izumi Asano, Carter Moravek, Yuichi Togashi, Hirohide Saito, Katsura Asano

**Affiliations:** 1 Center for iPS Cell Research and Application, Kyoto University, Sakyo-ku, Kyoto 606-8507, Japan.; 2 Graduate School of Science, Hiroshima University, Higashi-Hiroshima, Hiroshima 739-0046, Japan.; 3 RIKEN Center for Biosystems Dynamics Research (BDR), Wako, Saitama 351-0198, Japan.; 4 College of Life Sciences, Ritsumeikan University, Kusatsu, Shiga 525-8577, Japan.; 5 Molecular Cellular and Developmental Biology Program, Division of Biology, Kansas State University, Manhattan, KS 66506, USA.; 6 Graduate School of Integrated Sciences for Life, Hiroshima University, Higashi-Hiroshima, Hiroshima 739-8530, Japan.; 7 Research Center for the Mathematics on Chromatin Live Dynamics (RcMcD), Hiroshima University, Higashi-Hiroshima, Hiroshima, Japan 739-8530.; 8 RIKEN Center for Biosystems Dynamics Research (BDR), Higashi-Hiroshima, Hiroshima 739-0046, Japan.; 9 Hiroshima Research Center for Healthy Aging, Hiroshima University, Higashi-Hiroshima, Hiroshima 739-8530, Japan.

## Abstract

In contrast to prokaryotes wherein GUG and UUG are permissive start codons, initiation frequencies from non-AUG codons are generally low in eukaryotes, with CUG being considered as strongest. Here, we report that combined 5-cytosine methylation (5mC) and pseudouridylation (Ψ) of near-cognate non-AUG start codons convert GUG and UUG initiation strongly favored over CUG initiation in eukaryotic translation under a certain context. This prokaryotic-like preference is attributed to enhanced NUG initiation by Ψ in the second base and reduced CUG initiation by 5mC in the first base. Molecular dynamics simulation analysis of tRNA_i_^Met^ anticodon base pairing to the modified codons demonstrates that Ψ universally raises the affinity of codon:anticodon pairing within the ribosomal preinitiation complex through partially mitigating discrimination against non-AUG codons imposed by eukaryotic initiation factor 1. We propose that translational control by chemical modifications of start codon bases can offer a new layer of proteome diversity regulation and therapeutic mRNA technology.

## Teaser

Start codon choice can be recoded by chemical modification of its bases.

## INTRODUCTION

**Fig. 1.**
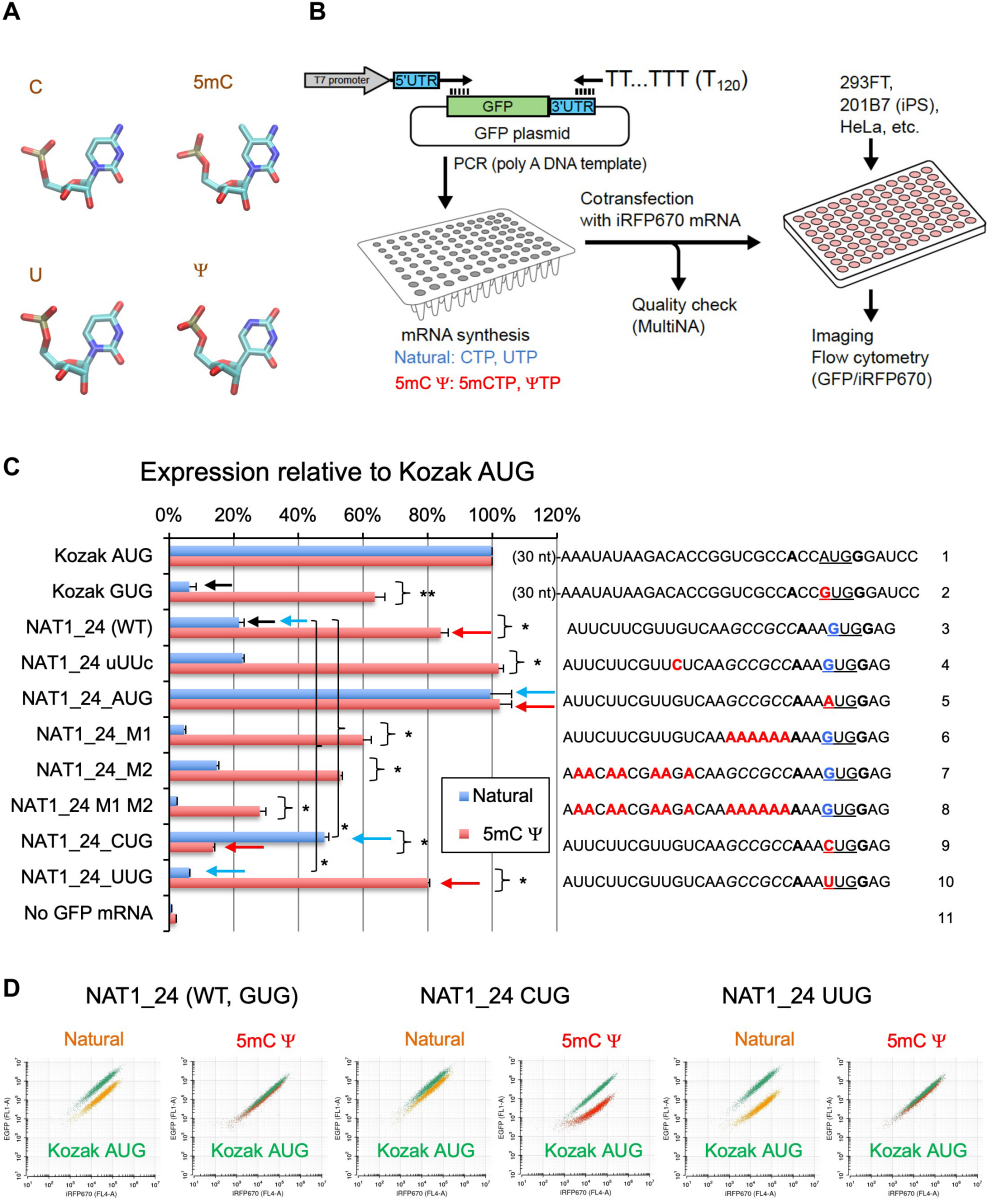
Double mRNA chemical modification alters start codon accuracy. (**A**) Atomic structure of chemically modified nucleotides (5mC and Ψ) is shown along with natural nucleotides C and U. Cyan, carbon; blue, nitrogen; red, oxygen; yellow, phosphorus. (**B**) Experimental scheme. Oligos containing wild-type or mutant versions of 5′UTR of NAT1/eIF4G2 or control synthetic *GFP* mRNA were synthesized to generate capped poly(A) *GFP* mRNA. The mRNA is cotransfected with *iRFP670* mRNA as internal standard. GFP/iRFP(670) expression ratio was quantified by flow cytometry. (**C**) Translation of natural or 5mC:Ψ-modified GFP mRNA with different non-AUG start codons was quantified in 293FT, relative to the value from *GFP* mRNA bearing the AUG start codon under a typical Kozak context (columns 1). 5′UTR nucleotide sequences of the constructs used in this figure are shown besides the graph. A (black) of the Kozak AUG codon or G (blue) of the first position of *NAT1* start codon was altered to G (red) or A, C, and U (red), respectively. Mutated residues in other constructs were also labeled red. Bars indicate SD (*n* = 3 except Kozak GUG, *n* = 2). *P < 0.003 and **P = 0.06 (*n* = 2). (**D**) The plot of GFP versus iRFP expression in 10,000 cells cotransfected with indicated *GFP* mRNAs and Kozak AUG *iRFP670* mRNA. For each mRNA shown, left shows the plot using natural mRNA (in orange), while the right, using double-modified mRNA (in red). For each panel, the plot using control Kozak AUG mRNA is shown in green.

In eukaryotes, mRNAs are chemically modified both terminally and internally. 5′-Terminal 7-methyl guanosine (m7G) capping and 3′-terminal polyadenylation are well known to promote translation initiation by the action of cytoplasmic cap-binding complex termed eukaryotic translation initiation factor 4F (eIF4F) and poly(A)-binding proteins, respectively (*1*, *2*). In contrast, eukaryotic mRNAs can be modified internally by 6-adenosine methylation, 5-cytosine methylation (5mC), hydroxymethylation (5hmC), pseudouridylation (Ψ) or N^1^-methylpseudouridylation (1mΨ), etc. (*3*). Despite plentiful reports on the role of 6-adenosine methylation in translational control (*1*, *4*), the roles of other mRNA modifications have not been well defined. In chemically synthesized mRNAs, 5mC and Ψ (Fig. 1A) have been extensively used for modifications in constructs used to express engineered proteins in vivo, for instance, for immunization, or to convert differentiated human cells to induced pluripotent stem cells (iPSCs) (*5*–*7*). The main purpose of introducing these modifications is to avoid innate immunity against foreign natural RNAs (*8*, *9*). With increasing evidence for natural occurrence of these modifications in human and other eukaryotic cells (*3*, *10*–*12*), we set out to examine whether 5mC and Ψ within the 5′ untranslated region (5′UTR) or the start codon affect translation efficiency. Of particular interest was their effect on near-cognate start codons such as GUG or CUG, because recent studies highlight some eukaryotic mRNAs displaying a high rate of initiation from such codons (*13*, *14*) that can be modified through 5mC or Ψ.

Prokaryotes (Archaea and Bacteria) use GUG and UUG start codons besides AUG (*4*, *15*). A regulatory role is proposed for some GUG or UUG codons, as they can be better sequestered and hence regulated by a small secondary structure when combined with a weak Shine-Dalgarno sequence (*16*, *17*). In contrast, non-AUG initiation generally occurs at a very low frequency in eukaryotes, because initiation from such codons is prevented by the actions of the components of eukaryote-specific multifactor initiation complex (*18*, *19*), with non-AUG discrimination role by eIF1 and AUG stabilizing role by eIF1A. Yet, certain nucleotide contexts and a secondary structure downstream of the codon allow strong non-AUG initiation, sometimes nearly strong as AUG codons (*13*, *14*, *20*). When compared under the same context, CUG is the strongest non-AUG start codon, while GUG and UUG follow in initiation strengths (*4*).

Recently, bacterial ribosome was found to decode ΨAG as a sense, rather than a stop codon, demonstrating an unexpected recoding capacity with chemically modified mRNAs in the A-site (*21*). Translational readthrough by chemically modified stop codons can allow modulating protein-coding capacity and may therefore offer an opportunity for technical innovation. However, it does not change the expression level of the targeted genes. In contrast, modulation of translation initiation by chemically modified start codons in the P-site may offer a greater impact on RNA technology if the modification can modulate the effect of eIFs on start codon selection and thereby alter the frequencies of initiation from various non-AUG start codons. Here, we show that the chemical modification of non-AUG start codon bases can alter the efficiency of translation initiation and present molecular dynamics (MD) simulation results, providing mechanistic details for this observation.

## RESULTS

### Experimental design

To search for mRNAs whose translation is affected by their chemical modification, we set up mRNA transfection assay as described in Fig. 1B. We first designed oligodeoxyribonucleotides corresponding to 5′UTR of any designed gene (table S1) and generated green fluorescent protein (GFP)–coding template for transcription driven by T7 RNA polymerase. In vitro transcription was conducted in the presence of chemically modified substrates in place of unmodified substrates, e.g., ΨTP and 5mCTP in place of uridine 5′-triphosphate (UTP) and cytidine 5′-triphosphate (CTP), respectively (fig. S1). The resulting mRNA products were used for lipo-transfection into cultured human cells [human embryonic kidney (HEK) 293FT, iPSC (201B7), and HeLa] and subsequent flow cytometry analysis for quantification of the *GFP* mRNA translation (fig. S2). As a control, we cotransfected *iRFP670* mRNA initiated by an AUG codon under a strong Kozak consensus. Translation efficiency was monitored by GFP expression normalized by the level of iRFP670 and presented as the value (%) relative to control *GFP* mRNA initiated by an AUG codon under a strong Kozak consensus (Fig. 1C, row 1, and fig. S2, A to C). Here, we report our finding using a subset of constructs derived from *NAT1/eIF4G2/DAP5* mRNA.

Human *NAT1/eIF4G2/DAP5* is one of the rare genes whose sole start codon is a near-cognate start codon, GUG (*22*–*24*). Previous expression study with cloned mRNA and its AUG-mutant version showed that the efficiency of the GUG initiation is about one-third of its AUG variant (*22*). We recapitulated the high rate of GUG initiation using a plasmid-borne luciferase reporter whose start codon is GUG and preceded by its original ~300-base-long 5′UTR, and *GFP* mRNA whose start codon is GUG and preceded by 24-base-long 5′UTR (*13*). Thus, we used *GFP* mRNA bearing 24- and 40-base-long *NAT1* 5′UTR to study the effect of 5mC or Ψ on non-AUG initiation (Fig. 1C, row 3, and figs. S2D, row 3, and S3A, row 1, blue bars).

To examine the effect of RNA modification on non-AUG start codons of various types and efficiencies, we used the following mutations altering *NAT1* 5′UTR. (i) The M1 mutation altering the rGCCGCC enhancer sequence located upstream of the *NAT1* start codon that substantially reduced translation of natural NAT1_24 mRNA (Fig. 1C, row 6, blue bar) (*13*). Of the six nucleotides of the enhancer context, the last C residue at the −4 position was recently verified to be conserved in strongly translated non-AUG start codons (*14*). (ii) The M2 mutation altering seven U residues located further upstream was newly generated for this study. This mutation slightly decreased *GFP* translation alone or in combination with M1 (*P* < 0.05 in 293FT; rows 7 and 8, blue bars), implicating a minor involvement of the altered area in *NAT1* translation. Because the level (~4 to 8% compared with AUG) of GUG initiation reduced by M1 or M1 M2 is equivalent to that of GUG initiation under a typical Kozak context (**A**CCGUG**G**) (*4*) (Fig. 1C and fig. S2D, row 2 versus row 6 or 8), the results with M1 and M1 M2, along with the GUG mutant version of control *GFP* mRNA, offer opportunities to test the effect on regular GUG initiation from a Kozak context. In contrast, those with M2 and WT *NAT1* offer to test the effect on GUG initiation from the enhancer context. (iii) The alteration of *NAT1* start codon to CUG that markedly increased translation and (iv) its alteration to UUG that decreased it significantly (Fig. 1C, rows 9 and 10, blue bars; fig. S3A, rows 4 and 5, blue bars; *P* < 0.005, *n* = 3) (*13*). Verifying a typical order of eukaryotic non-AUG start codon preference of AUG > CUG > GUG > UUG (*4*, *25*), these mutations offer opportunities to test the effect of two other major non-AUG start codons. (v) The variant altering a UUG codon located 16 nucleotides upstream of the *NAT1* start codon to UUC termed uUUc for upstream UUG to UUC. This was originally used to eliminate any possible effect of other NUG codon within the short UTR of NAT1_24 or NAT1_40. The involvement of this UUG codon was ruled out, as this mutation did not alter translation efficiency of *NAT1* mRNA (Fig. 1C, row 4, blue bars, and fig. S3A, row 2, blue bars). However, this mutation offered us an opportunity to test the reproducibility of the results obtained with WT *NAT1* constructs.

We previously showed that cellular degradation rate of *GFP* mRNAs was unaltered with or without the chemical modifications (5mC and Ψ), suggesting that the modification does not alter the stability of the control *GFP* mRNA under our experimental conditions (*26*). Translational repression of a derivative of the *GFP* mRNA (by an RNA binding protein to its 5′UTR) does not reduce mRNA abundance (*27*). Thus, by the direct measurement of GFP expression from tested mRNAs, we can evaluate the effect of chemical modifications on translation efficiencies from various start codons and 5′UTRs within the range of expression discussed here.

### Double modification by 5mC and Ψ markedly alters the efficiency of translation initiation from non-AUG codons

**Fig. 2.**
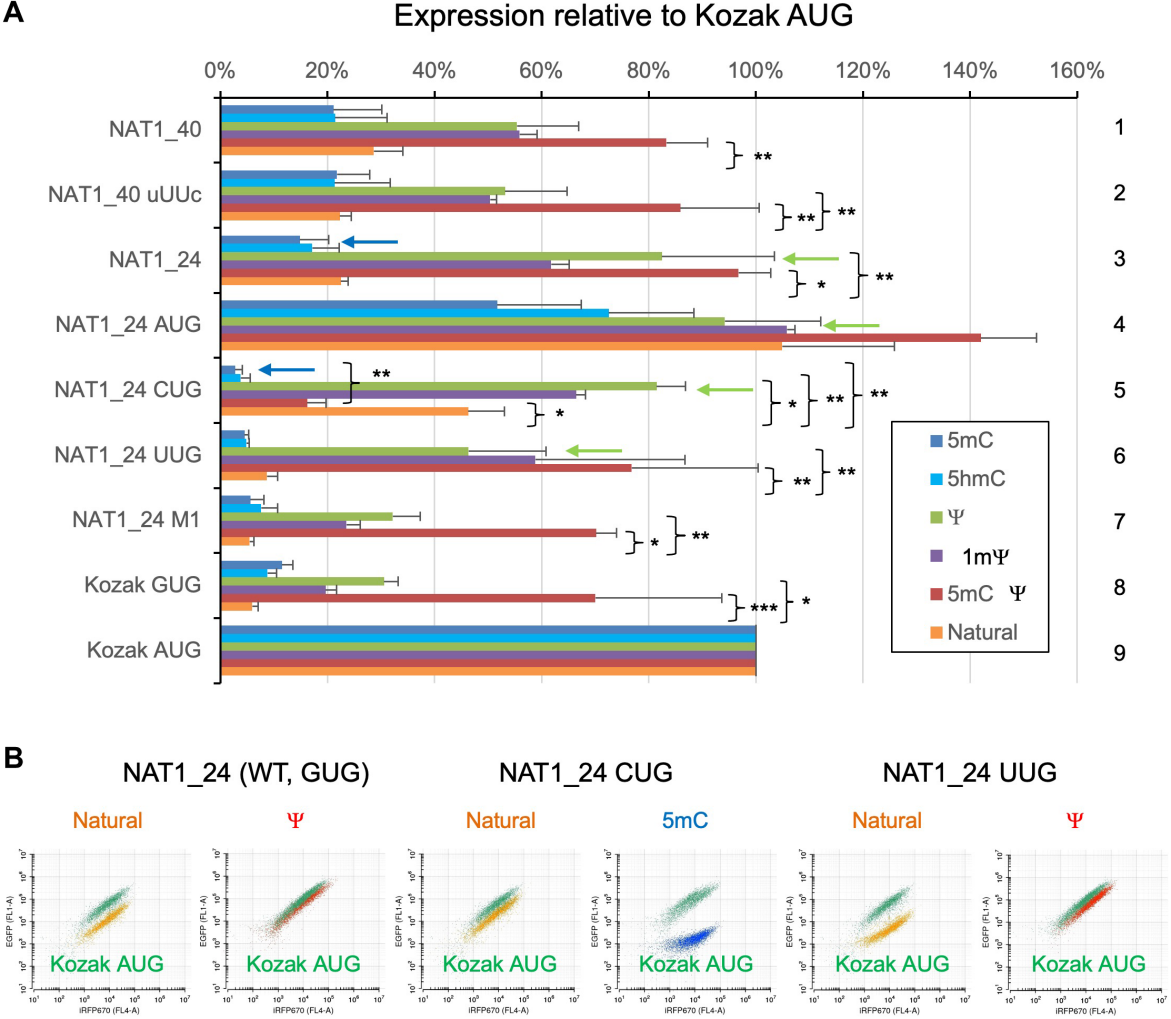
Effect of 5mC, 5hmC, Ψ, and 1mΨ on start codon specificity during translation initiation. (**A**) *GFP* mRNA derivatives with indicated 5′UTR were synthesized in the presence of 5mCTP (blue bars), 5hmCTP (cyan bars), ΨTP (green bars), 1mΨTP (purple bars), both 5mCTP and ΨTP (red bars), and unmodified nucleotides (orange bars) and subjected for expression assay. Median GFP/iRFP expression ratio was normalized to the value from Kozak AUG *GFP* mRNA with the same modification. Bars indicate SD (*n* = 3, except *n* = 2 for 1mΨ). *P < 0.01, **P ≤ 0.05, and ***P = 0.06. (**B**) The plot of GFP versus iRFP expression is shown for indicated mRNA species as in Fig. 1D; natural RNA in orange, specific modification in red or blue, and control mRNA in green.

The effect of the double modification by 5mC and Ψ (5mC:Ψ) is summarized in Fig. 1C and figs. S2 and S3. Figure 1D displays flow cytometry plots for typical experiments with *NAT1* mRNA initiated by GUG, CUG, and UUG codons. The 5mC:Ψ substantially increased GUG translation from all the tested *NAT1* or control mRNA variants to ~30 to 100% of the AUG initiation rate from the Kozak sequence (Fig. 1C, rows 2 to 4 and 6 to 8, or fig. S3A, rows 1 and 2, blue versus red bars). As a control, 5mC:Ψ displayed no significant effect on AUG versions of the same mRNA (Fig. 1C, rows 1 and 5, and fig. S3A, row 3). Thus, the 5mC:Ψ-induced changes likely stem from start codon context and not from other mRNA regions (however, see below). Similar to the GUG codon, 5mC:Ψ markedly increased translation from the UUG version of *NAT1* mRNAs (Fig. 1C, row 10, and fig. S3A, row 5). In contrast, 5mC:Ψ substantially decreased CUG initiation from the NAT1_24 or NAT1_40 CUG mRNAs (Fig. 1C, row 9, and fig. S3A, row 4). As a consequence, 5mC:Ψ altered NAT1 start codon preference to AUG > GUG ~ UUG > CUG similar to one observed with prokaryotic mRNAs (*15*). The same effect of 5mC:Ψ was observed in three different human cell lines: HEK293FT, iPSC 201B7, and cervical cancer HeLa cells (i.e., regardless of cells’ stemness or cancer) (figs. S2D and S3A, right; see below Fig. 2 for HeLa).

### Effect of individual chemical modification on non-AUG translation

To determine the impact of each chemical modification, we next introduced 5mC and Ψ separately into the reporter mRNAs (fig. S1). We confirmed that 5mC or Ψ alone did not exert a major effect on translation from the control Kozak AUG mRNA (fig. S3C). Experiments in HeLa cells with individually labeled mRNAs confirmed that the up-regulation of GUG initiation by 5mC:Ψ from mRNA with various 5′UTR sequences was due to the effect of Ψ (Fig. 2A, rows 1 to 3 and 7 to 9, green versus orange bars; see red for repeat in HeLa with 5mC:Ψ; see also Fig. 2B, left two graphs). Up-regulation of UUG initiation was also verified to be because of Ψ (Fig. 2, A, row 6, and B, right two graphs). In contrast, 5mC was responsible for strong repression of CUG initiation under the *NAT1* context (Fig. 2A, row 5, blue versus orange bars): The repression of this reporter expression by 5mC alone appears to be even stronger than the combined effect of 5mC:Ψ (row 5, blue versus red bars). This is likely due to the generally increasing effect of Ψ on initiation from NUG-type start codons. In agreement with this assessment, Ψ-modified version of the NAT1_CUG variant expressed more strongly than its nonmodified counterpart [Fig. 2, A (row 5, orange versus green bars) and B (middle two graphs)]. Together, these results indicate that Ψ in the second position of the GUG or CUG codons universally increases translation initiation regardless of the context, while 5mC in the first position of the CUG codon under the *NAT1* context decreases initiation (see below fig. S3B for effect on CUG under another context).

We also tested the effect of 1mΨ, which is used to avoid innate immunity [for example, by the Pfizer BioNTech SARS-CoV-2 mRNA vaccine Comirnaty (https://ema.europa.eu/en/medicines/human/EPAR/comirnaty)], or 5hmC, another common RNA modification in eukaryotes (*28*). Our results show that these modifications display an effect similar to Ψ or 5mC, respectively (Fig. 2A, cyan and purple bars), in agreement with their structural similarity to these nucleotides. We therefore focused our further studies on Ψ and 5mC.

### MD simulations provide insights into the mechanism of control of non-AUG translation by 5mC and Ψ

**Fig. 3.**
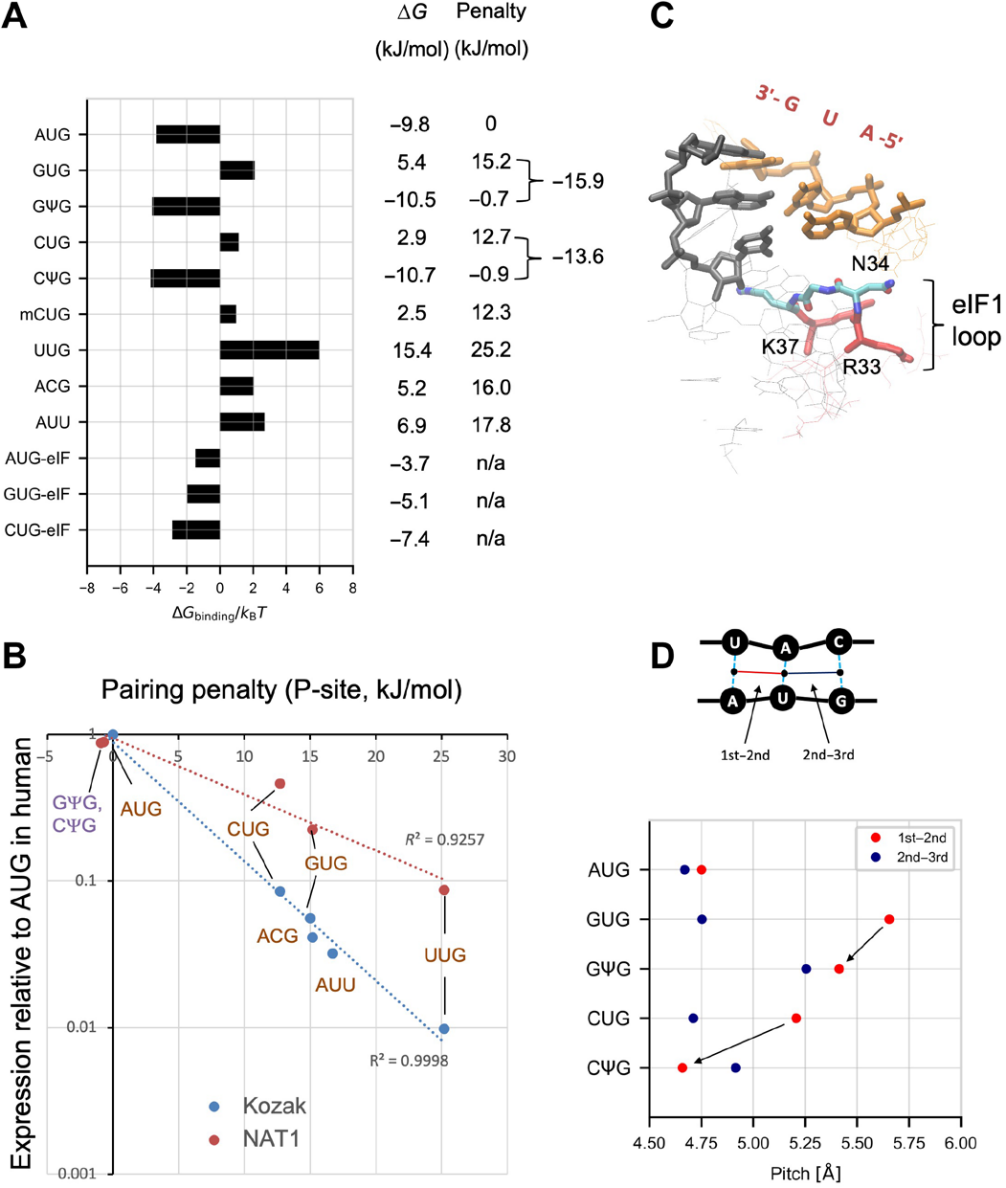
Determination of codon:anticodon affinity by the ABF method. (**A**) Estimated binding free energy. Δ*G*_binding_ score (see the schematics in fig. S4B) of all codons are shown. The scores were obtained from *P*(*d*_1_*, d*_2_*, d*_3_) averaged over five simulation trials for each model. Data for AUG, GUG, and CUG are taken from (*29*). Table to the right lists Δ*G*, and the energetic penalty compared with AUG. (**B**) Base pairing penalty relative to the free energy obtained for AUG in kJ/mol ($\Delta \Delta G={\Delta G}_{\text{binding}}-{\Delta G}_{\text{binding}}^{\text{AUG}}$) was plotted against initiation frequencies from indicated codons relative to one from AUG in *Homo sapiens* HEK293–derived cells, which was determined here using NAT1_24 mRNA derivatives (Fig. 1C) (orange circles) or previously using firefly luciferase reporters initiated by equivalent codons (blue circles) (*13*). (**C**) Average structure of the bound state $\left(\tilde{d_{1}},\tilde{d_{2}},\tilde{d_{3}})=(4.5,4.5,4.5\right)$ of eukaryotic P-site bearing AUG start codon (stick models in orange). Locations of anticodon (black) and eIF1 β-hairpin loop (stick model with atomic colorcode as in Fig. 1A except R33 and K37 in red) are highlighted. (**D**) Inter–base pair pitch calculated from the average structure of the bound state $\left(\tilde{d_{1}},\tilde{d_{2}},\tilde{d_{3}})=(4.5,4.5,4.5\right)$.

**Fig. 4.**
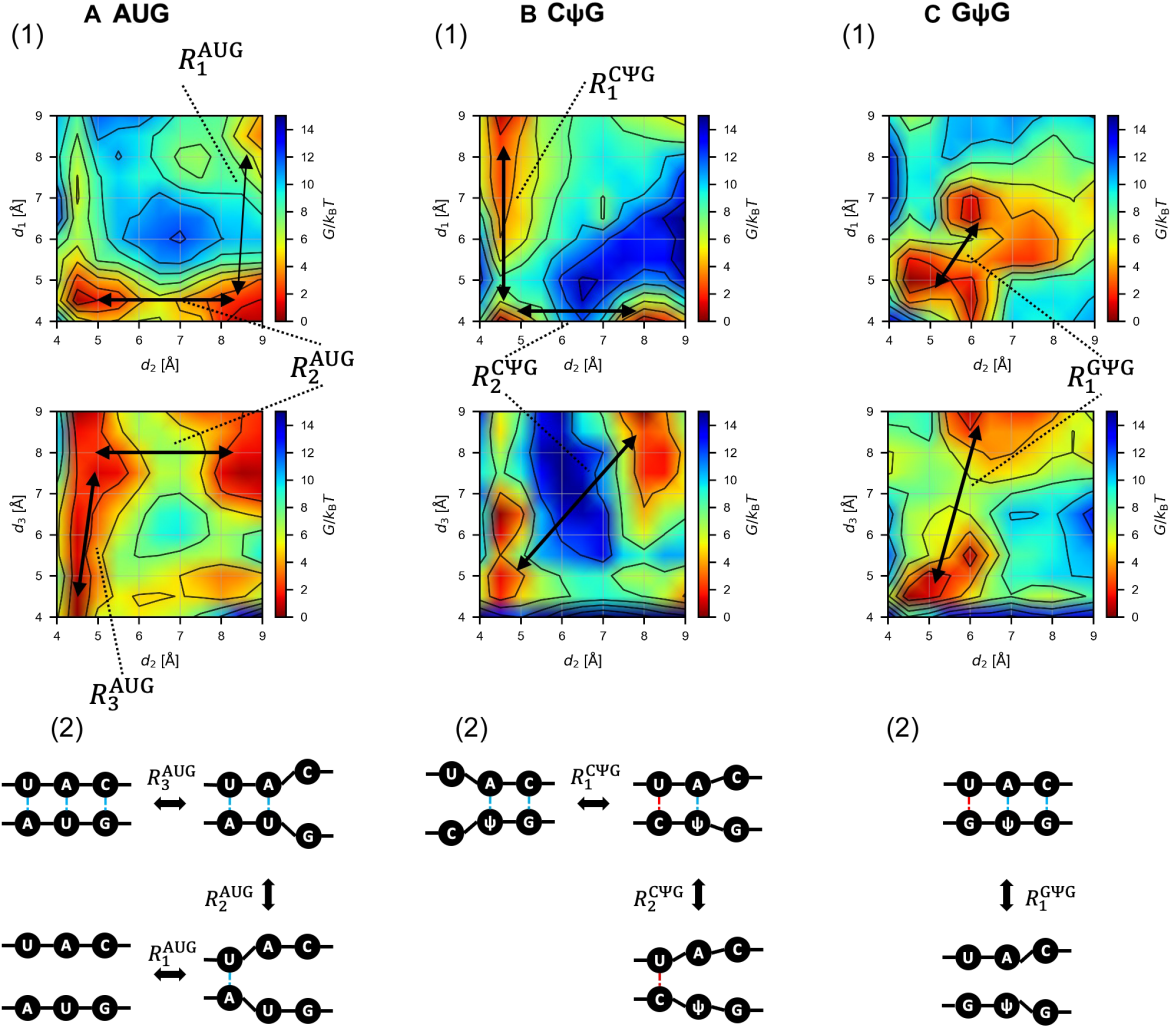
Schematics of the base pair binding dynamics. The base pairing dynamics are described for AUG (**A**), CΨG (**B**), or GΨG (**C**) start codons. Conformational changes inferred from the free energy landscape shown in the graphs in panel 1 (also see fig. S5) are summarized in panel 2. The transition path *R_n_^•^* (• is AUG, CΨG, or GΨG) is shown by black arrows. Dotted line, base-pairing distance; intermediate distance, close but not base pairing; large distance, weak or no interaction.

**Fig. 5.**
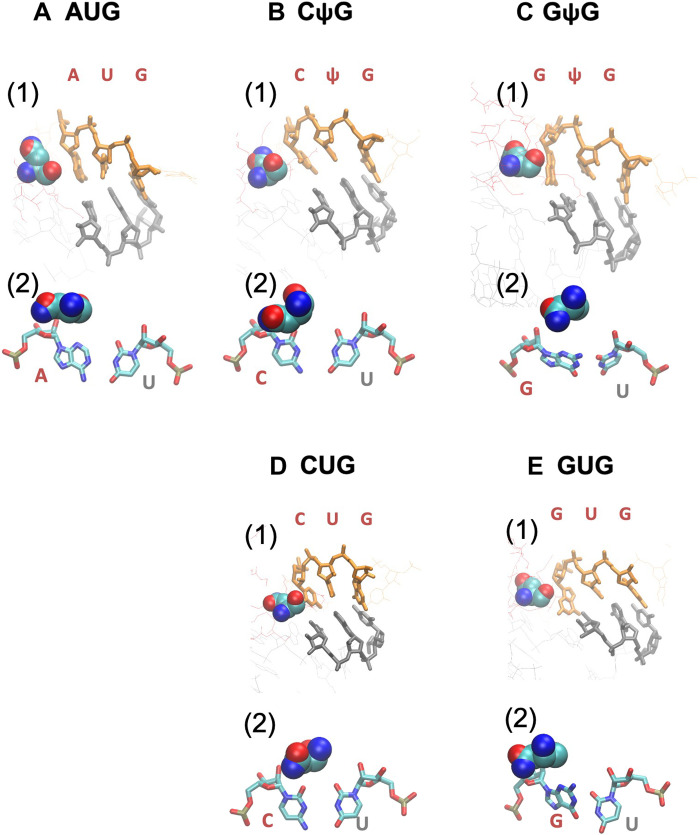
Average structures of start codon paired with anticodon in the P-site predicted by ABF MD simulation. Panels 1 and 2, averaged structures corresponding to $\left(\tilde{d_{1}},\tilde{d_{2}},\tilde{d_{3}})=(4.5,4.5,4.5\right)$ computed for AUG (**A**), CΨG (**B**), GΨG (**C**), CUG (**D**), and GUG (**E**) are presented. In panel 1, nucleotides of the codon (orange) and anticodon (gray) are drawn by thick lines. Thin red and blue lines are parts of eIF1 and eIF1A, respectively, with eIF1-N34 highlighted in a spherical model (with the same color code as Fig. 1A). In panel 2, the pair of bases at the first position is shown by stick models with the atomic color code as in Fig. 1A, along with the spherical model of eIF1-N34. In (A), (D), and (E), averaged structures corresponding to $\left(\tilde{d_{1}},\tilde{d_{2}},\tilde{d_{3}})=(4.5,4.5,4.5\right)$ are presented for AUG, CUG, and GUG, based on previously reported simulation study (*29*).

**Fig. 6.**
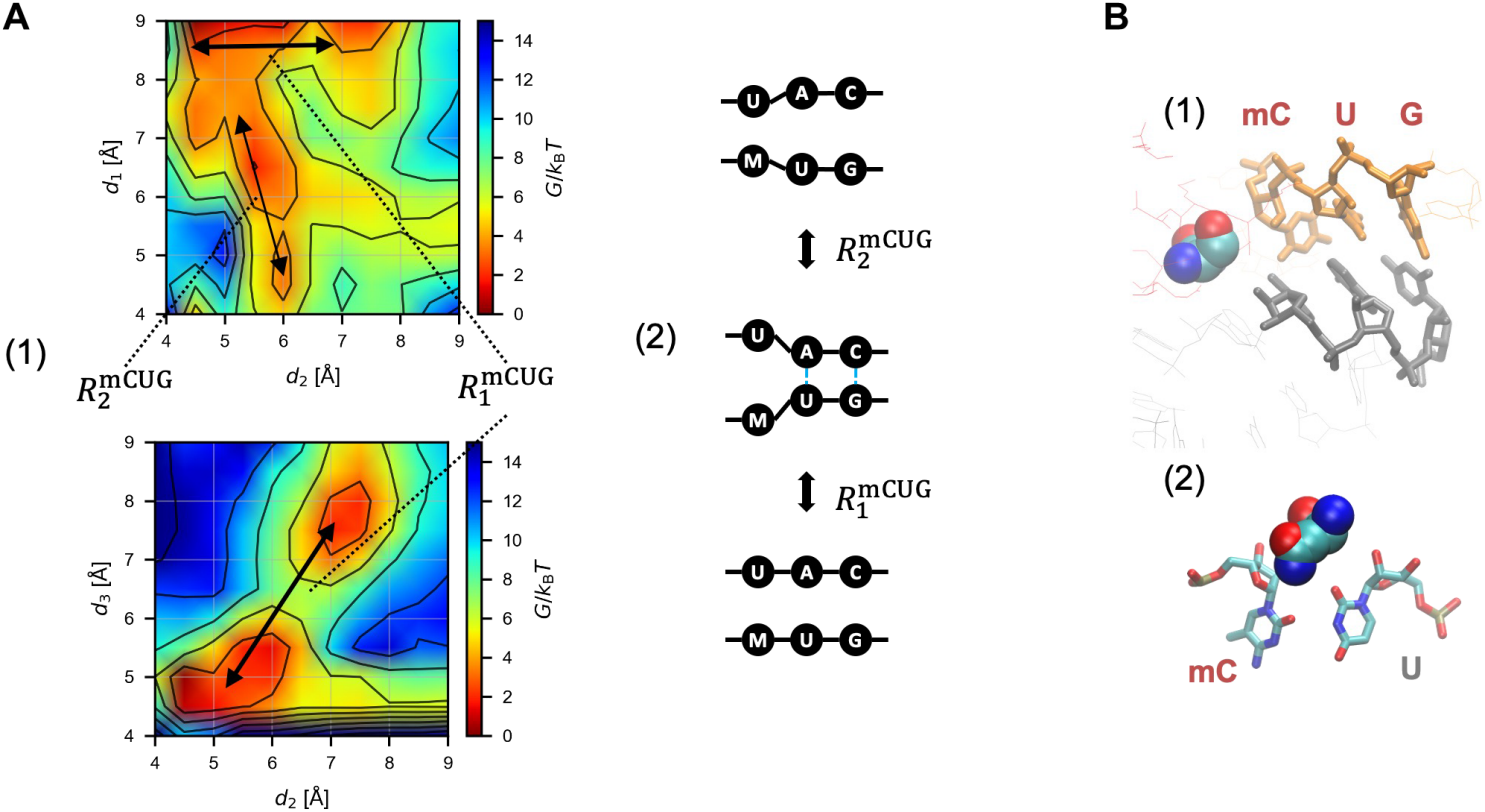
The mCUG pairing dynamics. (**A**) Base pair binding dynamics for mCUG. Panel 1, free energy landscape. Panel 2, deduced pairing pathway. (**B**) Average structure of the bound states of mCUG (orange) pairing to anticodon bases (gray) with eIF1 highlighted in panel 1 as in Fig. 5 (panel 1). Panel 2, the base pair at the first position.

To examine whether the observed effect of 5mC and Ψ is attributed to the effect on the start codon pairing stability in the ribosomal preinitiation complex (PIC), we performed MD simulations (*29*). We adopted the adaptive biasing force (ABF) method and evaluated free energy profiles (Figs. 3 to 6 and figs. S4 to S6) to elucidate the stability and the underlying mechanism in detail. We built an atomic model including the pairing of the tRNA_i_^Met^ anticodon CAU and the AUG start codon or near-cognate start codons within the *Saccharomyces cerevisiae* PIC structure (*30*), thereby examining the base-pairing stability in the context of eukaryotic PIC P-site. As shown in fig. S4A, the model includes mRNA with a start codon (table S2) and a part of eIF1 (see below), eIF1A, Met-tRNA_i_^Met^ anticodon loop, and rRNA. To evaluate the effect of mismatches at all three positions, we also examined ACG and AUU codons as a representative for mismatch at second and third positions. Using the results, first we evaluated the binding free energy Δ*G*_binding_ (fig. S4B). We then computed the base-pairing penalty for each start codon (ΔΔ*G*) by comparing to (subtracting with) the binding free energy obtained for AUG (${\Delta G}_{\text{binding}}^{\text{AUG}}$). As shown in Fig. 3A, the pairing stability of the unmodified near-cognate codons displayed reasonable values, as they correlated quite well with initiation frequencies from a Kozak context in human cells (*13*) (Fig. 3B, blue line), demonstrating its biological relevance. Ψ introduced to CUG and GUG markedly increased the pairing stability (Fig. 3A, parentheses) in agreement with increased expression from Ψ-modified mRNAs. Thus, the effect of Ψ on translation initiation from GUG or CUG codons of various mRNAs examined here likely results from increased selection of start codon at the P-site (see Fig. 3B, orange line, for correlation with GFP expression in Fig. 1).

Next, we examined the free energy profiles (Fig. 4, panel 1, and fig. S5). The results indicate stepwise base-pairing mechanisms as summarized in Fig. 4 (panel 2). The free energy landscape for the AUG pairing suggests the dissociation of the triplet base pairs starting at the third G-C base pair, likely through the tilted conformation of tRNA_i_^Met^ in the open PIC structure, with the affinity of the first A-U base pair likely increased by interaction with eIF1 (*29*). Likewise, CΨG pairing follows base-pairing pathway signifying stabilization at the first position base pair and dissociation at the third G-C pair, in agreement with the minor free energy difference. Of note was the proximity of its first C-U base pair when two other base pairs have dissociated (R_2_^CΨG^; Fig. 4B). However, the average structure of the bound state does not support C:U pairing at the first position (Fig. 5B, panel 2). Likewise, the GΨG pairing path was biphasic, without the G:U pair being observed outside of the fully bound state (Fig. 4C). These results strongly suggest that the Ψ:A pairing at the second position stabilizes the start codon recognition without fully impeding the discrimination against the GUG codon through eIF1 (*29*).

The discrimination against certain non-AUG codons through eIF1 has been proposed to be governed by its β-hairpin loop (RNGRK_33–37_) (*31*). This loop is located near the start codon with R33 and K37 (in red in Fig. 3C) being anchored to rRNA (*29*, *31*) [as R33A and K37E substitutions reduce the affinity against the 40*S* (*31*)]. Intriguingly, N34 is invariably located next to the first start codon base in all the examined structures (Fig. 5, A to E, and fig. S6, panel 2), apparently preventing the “up-shift” (as directed in Fig. 5, D and E, panel 2) of the first base (G or U) of the GUG or UUG codon, which otherwise allows wobble base pairing to the anticodon base U. Consequently, the first base of GUG or UUG is strongly displaced from the location to pair with the anticodon U (Fig. 5E and fig. S6C, panel 1), resulting in a mismatch at the first position even in the average “bound” structure. To consider the effect of eIF1 and eIF1A on eukaryotic start site selection, we removed eIF1 and eIF1A from the PIC model and recalculated Δ*G* for AUG, CUG, and GUG codons. As shown in Fig. 3A (bottom three rows), CUG and GUG pairings were markedly stabilized by the lack of these factors, consistent with eIF1’s role in discrimination against these codons. In contrast, AUG pairing was destabilized, in agreement with the role of (parts of) both these factors in promoting AUG initiation relative to non-AUG initiation (*32*–*34*).

The Ψ:A base pair was proposed to have higher stacking potentials compared with the U:A base pair (*35*). Thus, this trend could be proposed as the major driver of efficient selection of the NΨG start codon, generating the nucleation core of mismatched triplet base pairs while partially compensating for destabilization by eIF1. In agreement, the proposed CΨG and GΨG pairing pathways suggest that the base pairing is stabilized around the Ψ:A pair (Fig. 4, panel 2). Moreover, Ψ replacement of the second codon base shortens the distance between the first and second base pairs for CUG and GUG in the bound state (arrows in Fig. 3D; also compare Fig. 5, B and C, panel 1, versus D and E, panel 1). We therefore conclude that the stacking trend presented by the Ψ:A base pair is strong enough to allow the mismatched base pairs to form without fully resolving the aforementioned steric inhibition by eIF1-N34.

As shown in Fig. 6A (panels 1 and 2), the free energy landscape suggests that mCUG pairing is strongly prevented because of the mismatch at the first position. This is in contrast to CUG pairing for which no discernible path was observed (*29*), and the steric perturbation at the first base was not obvious (Fig. 5D). This, along with the average bound structure (Fig. 6B), strongly suggests that the methyl moiety prevents the positioning of the first codon base in the P-site. However, Δ*G* computed for mCUG was largely equivalent to that for CUG (Fig. 3A). We propose that this discrepancy is explained by mRNA context dependence. As shown in Fig. 3B, the expression value from CUG under the NAT1 enhancer context is an outlier in the relationship between the expression and the pairing stability. Thus, the NAT1 context is particularly suited for CUG initiation enhancement [which was verified by the dual-luciferease reporter assay (*13*)]. This context-dependent increase for the unmodified CUG is interpreted as being mitigated by the more universal destabilization mechanism through the 5-methyl addition. In agreement with the context dependence, 5mC:Ψ enhanced translation from a CUG start codon under a suboptimal Kozak context (fig. S3B, row 1); thus, Ψ in the second base seems to enhance translation, but 5mC in the first base apparently has little or no effect. The initiation from this CUG codon was verified by the CUG-to-CUC mutation, which diminishes expression from the reporter with 5mC:Ψ (fig. S3B, row 2). Alternatively, a possibility remains that the current ABF computational scheme cannot adequately evaluate the energetic effect of a small group, such as 5-methyl added to cytosine, on the codon:anticodon pairing in the P-site.

### Genetic evidence that eIF1-N34 is directly involved in discrimination against non-AUG codons

**Fig. 7.**
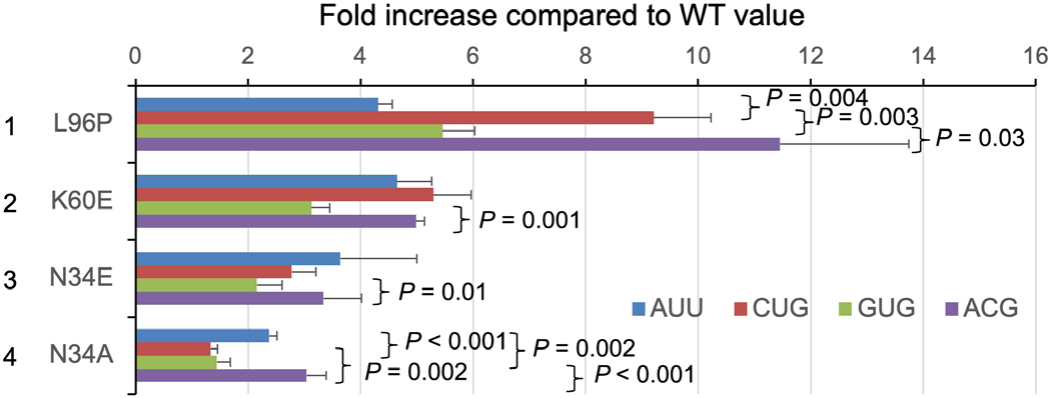
The possible role of eIF1-N34 in discrimination of the first codon base by size. Codon-specific effect of eIF1 mutations on initiation frequencies in yeast is presented in a graph. Fold increase compared with the average expression level relative to AUG in WT (see fig. S7) is presented for indicated eIF1 mutants. Yeast strains used are KAY1057 (*SUI1 LEU2 ura3*) and its isogenic derivatives, H4563 (*sui1-K60E*), H4564 (*sui1-L96P*), H4944 (*sui1-N34A*), and H4945 (*sui1-N34E*) (*19*, *31*). Bars indicate SEM. *P* values are shown for significant differences obtained with indicated pairs (experiments with eIF1-K60E, *n* = 8 for AUU and CUG and *n* = 6 for GUG and ACG; eIF1-L96P, *n* = 8 for each start codon; eIF1-N34E, *n* = 10 for CUG and GUG and *n* = 8 for AUU and ACG; eIF1-N34A, *n* = 14 for each start codon).

Having observed the proximity of eIF1-N34 to the first codon base and the suggested ability of the eukaryotic P-site to distinguish non-AUG codons by the size of its first base, we examined the effect of eIF1-N34 in vivo using yeast as a model. We paid attention to the yeast’s ability to distinguish adenine and cytosine at the first base of mismatched start codons. These two bases have common hydrogen donor/acceptor patterns, and the CCA-adding enzymes distinguish them merely by size for the nucleotide substrate binding (*36*). The caveat to this approach is that many eIF1 mutations reduce the affinity for the ribosome, thereby increasing non-AUG translation regardless of the codon type (suppressor of initiation codon mutation, or Sui^−^ phenotype) (*37*). Thus, we used eIF1-*K60E* and eIF1-*L96P* mutations defective in interaction with the 40*S* or with the 40*S* and eIF3c (*19*, *31*), respectively, as control. We used these and eIF1-*N34A* and eIF1-*N34E* mutants and examined initiation frequencies from GUG, CUG, ACG, and AUU start codons by luciferase reporter assays. As shown in fig. S7, all of these eIF1 substitutions increased frequencies from the four distinct codons, with eIF1-*L96P* bearing the strongest effects. As shown previously, GUG initiation is equivalent to or even higher than CUG or ACG initiation in yeast (*38*), suggesting that, along with UUG, the yeast system imposes a smaller penalty for GUG initiation, possibly through wobble pairing, for unknown reason. *K60E* increased frequencies from CUG, ACG, and AUU at similar magnitudes, greater than its effect on GUG initiation (Fig. 7, row 2). Thus, loose eIF1 association affects non-AUG codons equally, except GUG, whose initiation might be in part stabilized through wobble pairing. *L96P* essentially had the same effect as *K60E*, but its effect on AUU initiation was smaller as well (Fig. 7, row 1), suggesting a more preferential role for eIF3c binding in non-AUG codon repression at its first or second position. Of note, *N34A* more strongly increased ACG and AUU initiation than CUG initiation (Fig. 7, row 4), supporting the idea that eIF1-N34 can discriminate against adenine over cytosine at the first codon base, likely due to its size. *N34E* more strongly increased non-AUG initiation than *N34A* (fig. S7), as reported previously with a UUG codon reporter (*31*). Its overall trend of start codon derepression falls between those displayed by *K60E* and *N34A* (Fig. 7, row 3), suggesting that the specific effect of N34 substitution is confounded by the trend of minor eIF1 dissociation due to introducing an acidic amino acid. These results together suggest that N34 is used to distinguish cytosine over adenine at the first base of non-AUG start codons.

## DISCUSSION

Our ABF approach has been adapted to compute the binding free energy for codon-anticodon base pairing and is therefore not suited to evaluate energetic contribution from individual amino acids in the system. However, the examination of the most stable AUG paired structure (Fig. 3C) shows that the side chain of eIF1-N34 lies near the ribose moiety of the first codon nucleotide, with its peptide bond C═O projected against the codon base A. In contrast, bound-like structures for GUG pairing suggest that the C^6^═O of the first codon base G and the N34 peptide bond C═O are oriented away from each other. Thus, the N34 peptide bond orientation may at least, in part, account for a preference for adenine and cytosine (with NH_2_─ at C^6^ or C^4^, respectively) over guanine and uracil (with C═O at equivalent positions). The *N34A* mutation is, in turn, predicted to perturb the peptide bond orientation through a shorter alanine side chain less fit to bind the first codon nucleotide. The steric inhibition by eIF1-N34 is predicted to additionally confer fitness to a small base, generating the preference for cytosine over adenine (Fig. 7) or 5mC in a certain context (Figs. 2 and 6). More appropriate computational analyses focusing on protein contribution to initiation fidelity are warranted in the future.

In conclusion, we showed that, except for AUG, Ψ in the second NUG codon base strongly enhances initiation through increased base pairing to the mismatched start codon, while 5mC in the first base of a CUG codon diminishes initiation in a context-dependent manner. While non-AUG translation is known to be regulated by trans-acting factors independently of the codon types (*13*, *25*, *39*), codon-specific regulation has not been reported. To our knowledge, this is the first report that shows that the chemical modification of near-cognate start codons can specifically alter initiation frequency and hence recode translation initiation. The ABF approach was proven to serve as a powerful tool to study base-pairing dynamics in translation initiation. Technically, our finding broadens the repertoire for the application of mRNA technology to in vivo therapeutic expression. For instance, NΨG-initiated expression therapy from synthetic mRNA would markedly reduce the chance for unwanted expression from a rare cDNA product that could integrate into the host genome, allowing a clean effect from the administrated mRNA molecules.

## MATERIALS AND METHODS

### Preparation of *GFP* and *iRFP670* mRNA

DNA template for *GFP* mRNA was generated by polymerase chain reaction (PCR) using mRNA-specific 5′-oligos and the common 3′-oligo, all listed in table S1. Plasmid pUC19–EGFPfull carrying the enhanced GFP coding sequence and primer-binding sites (Y.F., personal stock) was used as template. PCR was set up using KOD plus ver.2 (Toyobo, Japan) (94°C for 2 min, 20 cycles of 98°C for 10 s, 60°C for 30 s, and 68°C for 1 min and, lastly, 15°C forever) and purified by MiniElute PCR purification Kit (Qiagen). DNA template for *iRFP670* mRNA was generated similarly using as template the coding region of iRFP670 in piRFP670-N1 (Addgene plasmid no. 45457) (*40*).

In vitro transcription was conducted with the resulting DNA using MEGAscript kit (Ambion) supplemented with anti-reverse cap analog (TriLink BioTechnologies). To uniformly modify mRNAs, we used the following chemically modified nucleotides in place of equivalent nucleotides (TriLink BioTechnologies) at 7.5 mM (the same concentration as specified for CTP and UTP by the manufacturer); 5-methylcytidine-5′-triphosphate (10 μmol, N-1014-10), 5-hydroxymethylcytidine-5′-triphosphate (5 μmol, N-1087-5), 1-methylpseudouridine-5′-triphosphate (10 μmol, N-1081-10), and pseudouridine-5′-triphosphate (10 μmol, N1019-10).

The transcribed mRNAs were treated with TURBO DNase (Ambion) and rAPid alkaline phosphatase (Roche), followed by purification using FavorPrep total RNA extraction column (Favorgen). The mRNA products were analyzed by the MultiNA microchip electrophoresis system (Shimadzu). RNA concentration was determined by NanoDrop (Thermo Fisher Scientific). The average of the three measurements was used to make RNA solution at a fixed concentration.

### GFP mRNA translation assay

Equal amounts (20 ng) of mRNAs coding for GFP or iRFP670 were cotransfected to cells, which were seeded in a 96-well plate at 2 × 10^4^ cells per well on the day before the day of transfection. Transfection used Stemfect (Stemgent) for 201B7 and Lipofectamine 2000 (Thermo Fisher Scientific) for 293FT and HeLa. Fluorescence images of the transfected cells were captured on the RS100 automated imaging system (Olympus) on 1 day after transfection. Then, the cells were washed by phosphate-buffered saline once and treated with Accumax (Innovative Cell Technologies) at 37°C for 10 min. The detached cells were analyzed by Accuri C6 using FL1 (533/30 nm) and FL4 (675/25 nm) for GFP and iRFP670, respectively. Flow cytometry data were analyzed using R with flowCore packages (*41*). Live and iRFP670-positive cells were gated, and then median of ratio of GFP/iRFP670 of individual cells was calculated and defined as translational efficiency.

### Dual-luciferase assay

pSV40 AUG-Fluc or its non-AUG start codon derivative was cotransfected with pSV40 AUG-Rluc in HEK293T, and the transfectants were subjected for Dual Glo^R^ luciferase assay (Promega), all as described in (*13*, *25*). For assays in yeast *S. cerevisiae*, transformants of appropriate yeast strains bearing pFluc_AUG_ Rluc_AUG_ (*URA3*) or its Fluc non-AUG start codon derivative (*38*) were grown in synthetic complete media lacking uracil (SC-ura) at 30°C for 4 to 6 hours to an exponential phase and placed on ice. 0.075 A_600_ unit in 8 μl was collected by centrifugation and loaded in duplicate to the 96-well assay plate. After 30 min of incubation with the Dual Glo reagent (8 μl) (Promega), luminescence was measured (for Fluc) in Victor 3 (Perkin Elmer), followed by treatment with the addition of 8 μl of Stop-and-glo reagent (Promega) and another measurement (for Rluc) in 10 min.

### Estimation of free energy landscape by the ABF method

For the analysis of the codon-anticodon interaction in the eukaryotic ribosomal PIC, we adopted a cryo-EM structure of the yeast 48S PIC (Protein Data Bank ID: 3J81) (*30*). We extracted atoms located within 25 Å from N1 atom in the middle base of anticodon in the tRNA molecule (*29*, *42*). Then, the bases were substituted when necessary, and missing atoms were supplemented to construct PIC models involving target codons including chemically modified codons (table S2). Models without eIF1 and eIF1A were also prepared for the AUG, GUG, and CUG cases. Last, the model was enclosed in a 36-Å-radius sphere of TIP3P water with 150 mM KCl. After energy minimization and equilibration (10 ns), we performed MD simulation with the ABF method (1 μs), five trials for each case. Temperature and pressure were set at 310 K and 1 atm.

In the ABF MD simulation (*43*), the distances *d*_1_, *d*_2_, and *d*_3_ of the first, second, and third base pairs in angstrom unit, respectively (table S2), were adopted as the conformational coordinates. Each *d_i_* was sampled over 4.0 ≤ *d_i_* ≤ 9.0 with bin-width ∆*d* = 0.5, to obtain the probability *P*(*d*_1_, *d*_2_, *d*_3_) of each conformational state. The Gibbs free energy was calculated as *G*(*d*_1_, *d*_2_, *d*_3_) = − *k*_B_*T* ln *P*(*d*_1_, *d*_2_, *d*_3_) + const., which is further reduced to two dimensions as *G*(*d*_1_, *d*_2_), *G*(*d*_1_, *d*_3_), and *G*(*d*_2_, *d*_3_) (Fig. 4 and fig. S5). The binding free energy was evaluated as ∆*G*_binding_ = *G*_bound_ − *G*_unbound_, the difference of the free energy of the bound and unbound states defined as 4.0 ≤ *d*_1_, *d*_2_, *d*_3_ ≤ 6.0 and 7.0 ≤ *d*_1_, *d*_2_, *d*_3_ ≤ 9.0, respectively (fig. S4B). To evaluate the convergence of the probability, the squared error function *L*(τ_1_, τ_2_) = Σ_4.0 ≤ *d*_1_, *d*_2_, *d*_3_ ≤ 9.0_[*P*(*d*_1_, *d*_2_, *d*_3_; τ_1_) − *P*(*d*_1_, *d*_2_, *d*_3_; τ_2_)]^2^ between two time points was used. The averaged structure for representative coordinate $\left(\tilde{d_{1}},\tilde{d_{2}},\tilde{d_{3}}\right)$ was reconstructed by averaging each atomic coordinate over all the snapshots satisfying $\forall i:\tilde{d_{i}}-\frac{\text{∆}d}{2}\leq d_{i}\leq \tilde{d_{i}}+\frac{\text{∆}d}{2}$. The position of each base pair was represented by the center of the two C1′ atoms in the averaged structure, and the inter–base pair pitch was calculated as the distance between them. As the averaging is applied independently to each atomic coordinate, the structure can be skewed in regions where conformational fluctuations are large, particularly where free rotation around the bonds is possible (e.g., in Figs. 3C and 5, panel 2).

Throughout the modeling and simulation, CHARMM36 force field (July 2019) was applied. VMD was used for modeling and visualization (*44*), and NAMD (version 2.13 multicore) was used for the simulation (*45*). All the other protocols and parameters were the same as those in (*29*); for details, see Materials and Methods therein.

## Notes

View/request a protocol for this paper from *Bio-protocol*.
