# Supplementary material for: Translational recoding by chemical modification of non-AUG start codon ribonucleotide bases
Source: Sci Adv. 2022 Apr 8;8(14):eabm8501. doi: 10.1126/sciadv.abm8501 (PMC11706245; doi:10.1126/sciadv.abm8501)
Supplement: Supplementary file 1 — Tables S1 and S2 Figs. S1 to S7 [file sciadv.abm8501_sm.pdf]

Science Advances
AAAS

Supplementary Materials for

Translational recoding by chemical modification of non-AUG start codon ribonucleotide bases

Yoshihiko Fujita, Takeru Kameda, Chingakham Ranjit Singh, Whitney Pepper, Ariana Cecil, Madelyn Hilgers, Mackenzie Thornton, Izumi Asano, Carter Moravek, Yuichi Togashi*, Hirohide Saito*, Katsura Asano*

*Corresponding author. Email: togashi@fc.ritsumei.ac.jp (Y.T.); hirohide.saito@cira.kyoto-u.ac.jp (H.S.); kasano@ksu.edu (K.A.)

Published 8 April 2022, *Sci. Adv.* **8**, eabm8501 (2022)
DOI: 10.1126/sciadv.abm8501

This PDF file includes:

Tables S1 and S2
Figs. S1 to S7

Table S1. Oligodeoxyribonucleotides used for GFP mRNA production.

Oligo Name	mRNA Name	Sequence
KA002_hNAT1_2	NAT1_uCUG	CGCTAATACGACTCACTATAGGGctgcagcagcgactcctctgagCTGagtGTGAGCAAGGGCGAGGAG
KA003_hNAT1_3	NAT1_40	CGCTAATACGACTCACTATAGGGtattattcttttgaagattcttcgTTGtcaagccgccaaaGTGgagGTGAGCAAGGGCGAGGAG
KA028-hNAT1_2a	NAT1_uCUc	CGCTAATACGACTCACTATAGGGCTGCAGCAGCGACTCCTCTGAGCTcAGTGTGAGCAAGGGCGAGGAG
KA029-hNAT1_3a	NAT1_40 uUUc	CGCTAATACGACTCACTATAGGGTATTATTCTTTTGAAGATTCTTCGTTcTCAAGCCGCCAAAGTGGAGGTGAGCAAGGGCGAGGAG
KA055-hNAT1_3c	NAT1_40_AUG	CGCTAATACGACTCACTATAGGGTATTATTCTTTTGAAGATTCTTCGTTGTCAAGCCGCCAAAATGGAGGTGAGCAAGGGCGAGGAG
KA059-hNAT1_3d	NAT1_40_CUG	CGCTAATACGACTCACTATAGGGTATTATTCTTTTGAAGATTCTTCGTTGTCAAGCCGCCAAACTGGAGGTGAGCAAGGGCGAGGAG
KA060-hNAT1_3e	NAT1_40_UUG	CGCTAATACGACTCACTATAGGGTATTATTCTTTTGAAGATTCTTCGTTGTCAAGCCGCCAAATTGGAGGTGAGCAAGGGCGAGGAG
KA065_NAT1_24_WT	NAT1_24 (WT)	CGCTAATACGACTCACTATAGGATTCTTCGTTGTCAAGCCGCCAAAGTGGAGGTGAGCAAGGGCGAGGAGCTGTTC
KA066_NAT1_24_AUG	NAT1_24_AUG	CGCTAATACGACTCACTATAGGATTCTTCGTTGTCAAGCCGCCAAAATGGAGGTGAGCAAGGGCGAGGAGCTGTTC
KA067_NAT1_24_M1	NAT1_24_M1	CGCTAATACGACTCACTATAGGATTCTTCGTTGTCAAAAAAAAAAGTGGAGGTGAGCAAGGGCGAGGAGCTGTTC
KA068	NAT1_24_M2	CGCTAATACGACTCACTATAGGAAACAACGAAGACAAGCCGCCAAAGTGGAGGTGAGCAAGGGCGAGGAGCTGTTC
KA072-NAT1-24-CUG	NAT1_24_CUG	CGCTAATACGACTCACTATAGGATTCTTCGTTGTCAAGCCGCCAAACTGGAGGTGAGCAAGGGCGAGGAGCTGTTC
KA073_NAT1_24_UUG	NAT1_24_UUG	CGCTAATACGACTCACTATAGGATTCTTCGTTGTCAAGCCGCCAAATTGGAGGTGAGCAAGGGCGAGGAGCTGTTC
KA074	NAT1_24 uUUc	CGCTAATACGACTCACTATAGGATTCTTCGTTCTCAAGCCGCCAAAGTGGAGGTGAGCAAGGGCGAGGAGCTGTTC

KA075	NAT1_24 M1 M2	CGCTAATACGACTCACTATAGGAAACAACGAAGACAAAAAAAAAAAGTGGAGGTGAGCAAGGGCGAGGAGCTGTTC
KA077-GTG-EGFP-Fwd	Kozak GUG	TTGTAATACGACTCactatagggcgaattaagagagaaaagaagagtaagaagaaatataagacaccggtcgccaccGtgggatccgtgagcaagggcga
SKC111_TAP_T7_G3C	Kozak AUG	CAGTGAATTGTAATACGACTCACTATAGGGC
KEC883_3UTR120A	Universal 3'-end primer	TTTTTTTTTTTTTTTTTTTTTTTTTTTTTTTTTTTTTTTTTTTTTTTTTTTTTTTTTTTTTTTTTTTTTTTTTTTTTTTTTTTTTTTTTTTTTTTTTTTTTTTTTTTTTTTTTTTTTTTTCCTACTCAGGCTTTATTCA

Table S2. Codons of focus in our simulation. Nucleotide compositions and atom selection to determine the position to define the distance of matchable base pair. The center of the listed atoms is used as the position of the nucleotide to measure distances.

**Codon**	**Front Nucleotide**		**Middle Nucleotide**		**Prior Nucleotide**	
**AUG**	A	N1 and N6	U	N3 and O4	G	N1, N2, and O6
**GUG**	G	N1, N2, and O6	U	N3 and O4	G	N1, N2, and O6
**GΨG**	G	N1, N2, and O6	Ψ	N3 and O2	G	N1, N2, and O6
**CUG**	C	N3, N4, and O2	U	N3 and O4	G	N1, N2, and O6
**CΨG**	C	N3, N4, and O2	Ψ	N3 and O2	G	N1, N2, and O6
**mCUG**	5mC	N3, N4, and O2	U	N3 and O4	G	N1, N2, and O6
**UUG**	U	N3 and O4	U	N3 and O4	G	N1, N2, and O6
**ACG**	A	N1 and N6	C	N3, N4, and O2	G	N1, N2, and O6
**AUU**	A	N1 and N6	U	N3 and O4	U	N3 and O4

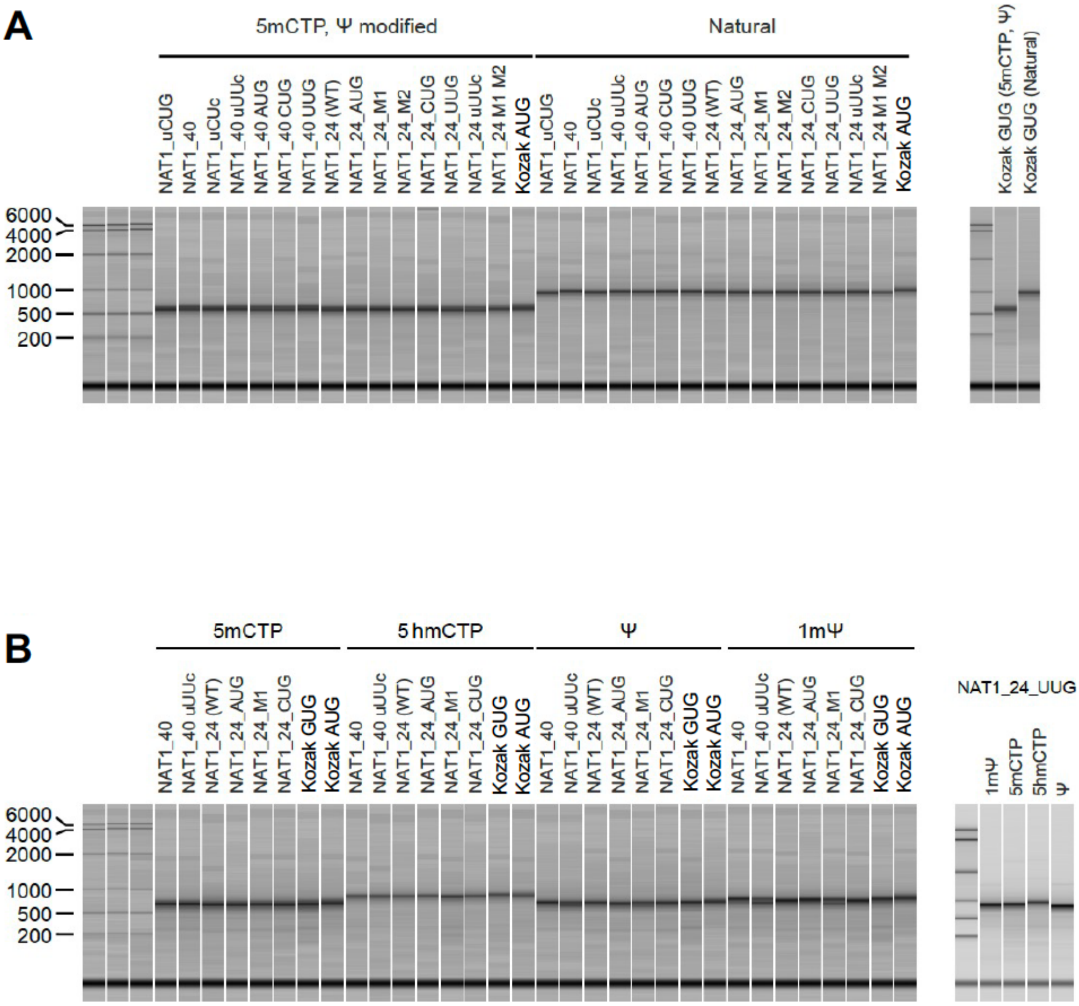

Figure S1. GFP reporter mRNAs used in this study. ***Related to Figs. 1 and 2.*** Electrophoretic patterns from MultiNA (Shimadzu) for natural, 5mC:Ψ- (A) or individually (B) modified mRNA are shown with size standards.

Fujita et al. Fig. S2

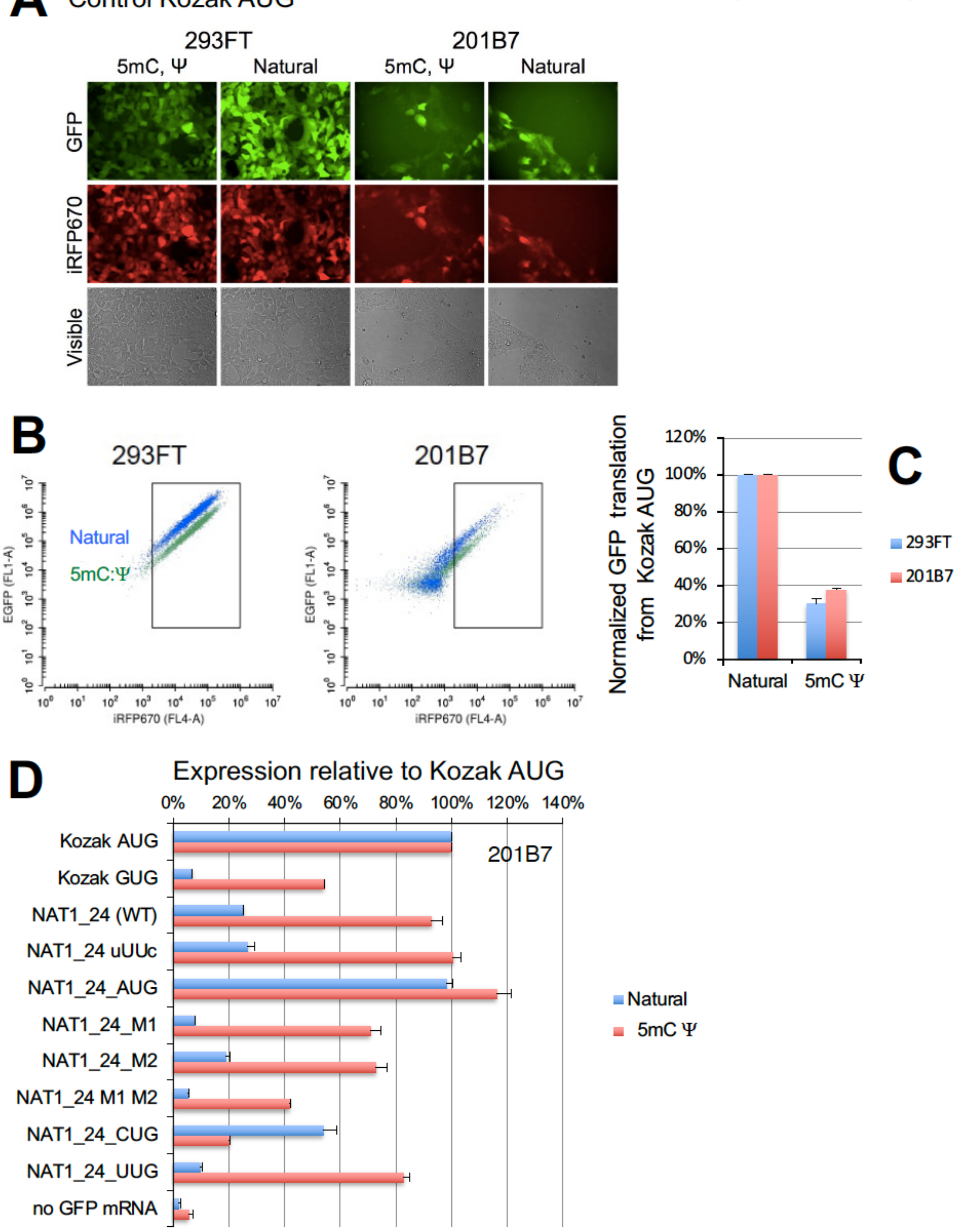

Figure S2. GFP mRNA translation assay. ***Related to Fig. 1.*** (A) Pictures of 293FT or 201B7 cells co-transfected with *iRFP670* mRNA and natural or 5mC:Ψ-modified version of control Kozak AUG mRNA were taken under visible light or green or red fluorescence. (B) GFP and iRFP signals from 10,000 cells were plotted for control GFP mRNA (natural, blue; 5mC:Ψ, green). (C) *GFP* expression from 5mC:Ψ-modified Kozak AUG mRNA was compared to that from natural Kozak AUG mRNA in 293FT

(n=3) and 201B7 (n=2). Error bars indicate SD. (D) Translation of natural or 5mC:Ψ-modified *GFP* mRNA with different non-AUG start codons was quantified in human iPSC 201B7, as in Fig. 1C. Error bars indicate SD (n=2).

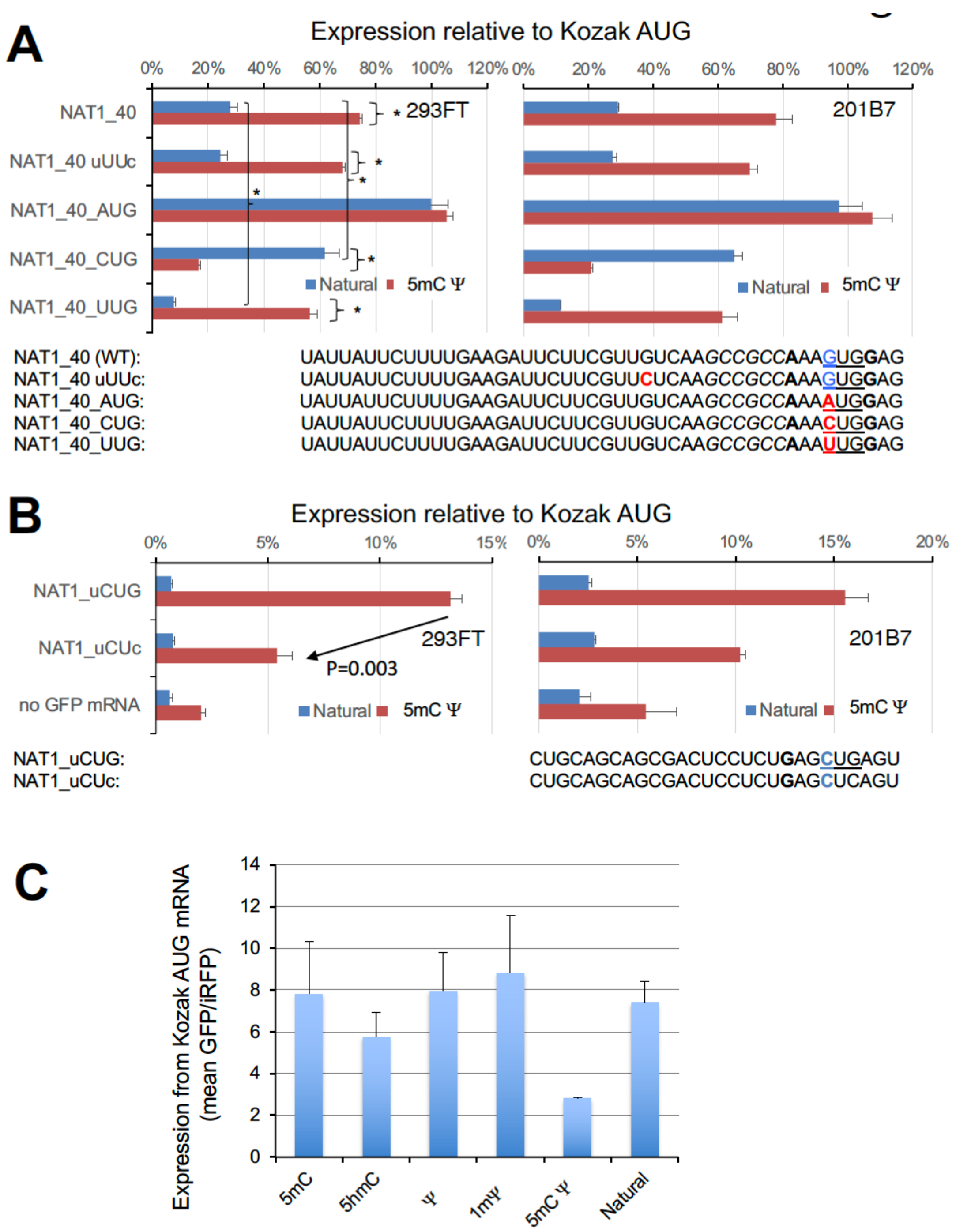

Figure S3. Additional GFP mRNA translation assay. ***Related to Figs. 1 and 2.*** (A) and (B) Translation of natural or 5mC:Ψ-modified GFP mRNA with additional non-AUG constructs was quantified in reference to co-transfected iRFP mRNA in 293FT (left, n=3) and 201B7 (right, n=2) and presented as in Fig. 1C. The nucleotide sequences of 5' UTR of indicated constructs are shown below the graphs. Bars indicate SD. *, $p<0.01$ (n=3). (C) Effect of chemical modifications on Kozak AUG translation. Indicated versions of control Kozak AUG mRNA was co-transfected into HeLa with iRFP670

mRNA and GFP/iRFP ratio was computed after FACS analysis. GFP/iRFP ratios in arbitrary units were shown from two independent experiments with bars indicating SD (n=3).

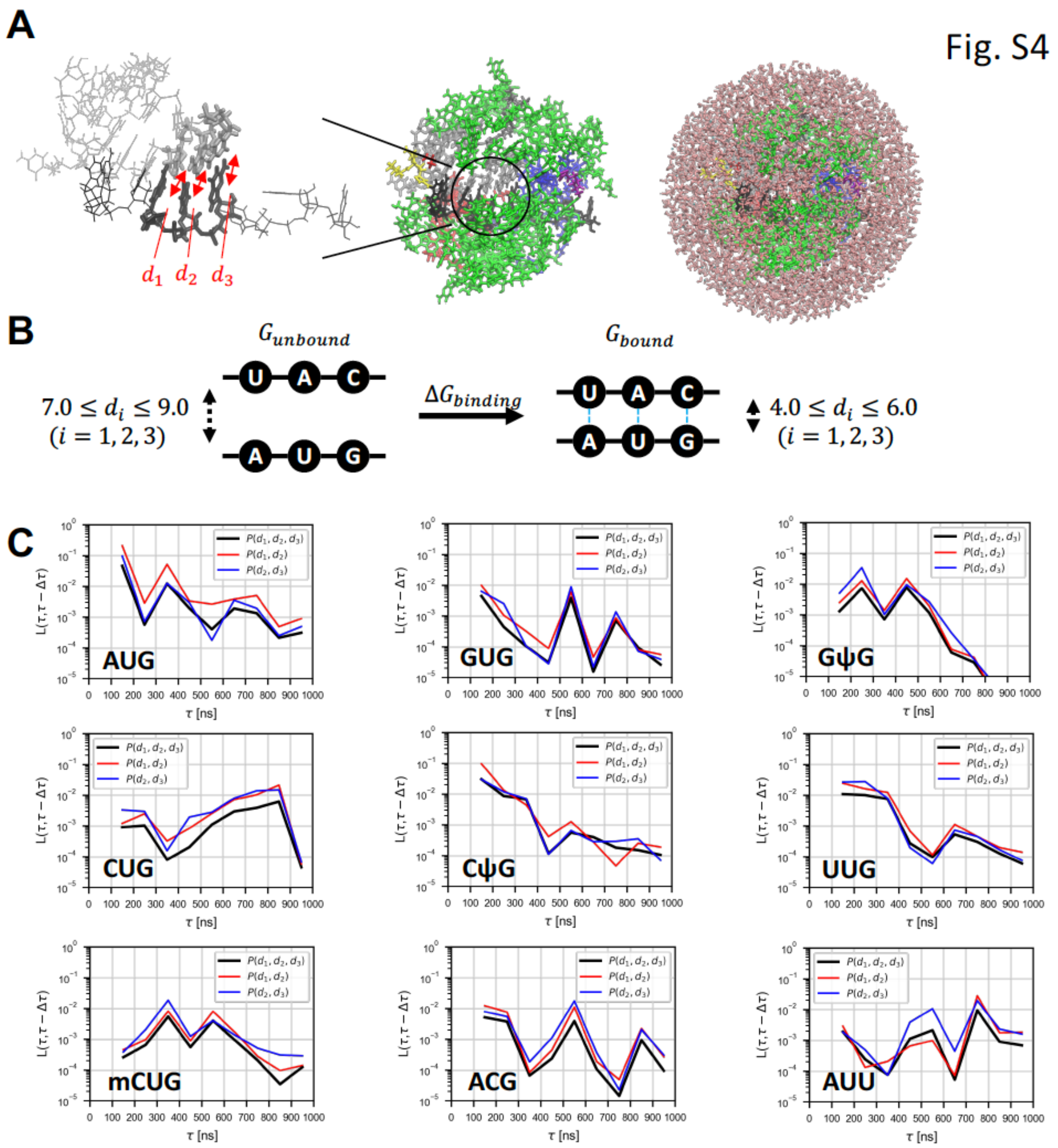

Figure S4. MD simulations of codon: anticodon interactions in the eukaryotic P site by the ABF method. ***Related to Fig 3.*** (A) The PIC model and reaction coordinates. (Left) Only structures of mRNA and tRNA around three matchable base pairs (Table S2) are shown in black and silver. Three individual distances d_1, d_2, and d_3 are shown. (Center) rRNA segments (split) are shown in green, and protein components are shown in other colors. (Right) Water molecules are shown in pink, which enclose the system and form a sphere. (B) Schematics of G_{bound}, G_{unbound}, and $\Delta G_{\text{binding}}$. AUG codon is used to visualize the bound and unbound states (adapted from (*29*)). (C) Convergence of free energy profiles with time evolution. The $L(\tau, \tau - 100.0)$ of $P(d_1, d_2, d_3)$, $P(d_1, d_2)$, and $P(d_2, d_3)$ are shown.

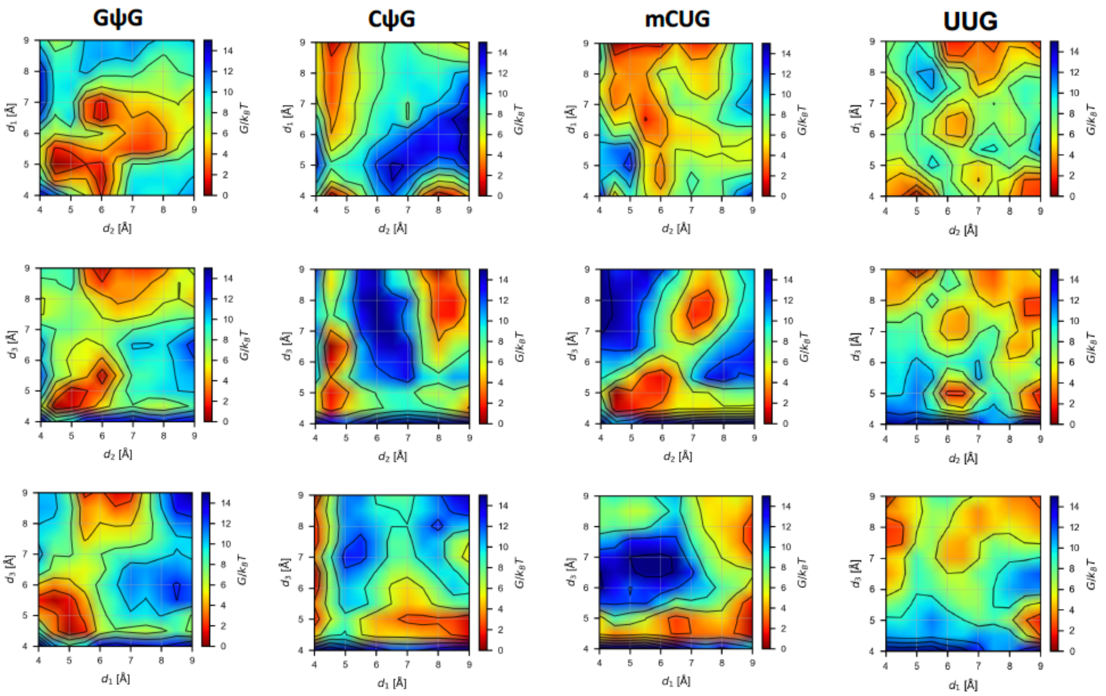

Figure S5. Projected free energy profiles. ***Related to Fig 4 and 6.*** Profiles of $G(d_1, d_2)$, $G(d_2, d_3)$, and $G(d_1, d_3)$ (see Methods) for indicated start codon with the PIC P-site model are shown by contour plots.

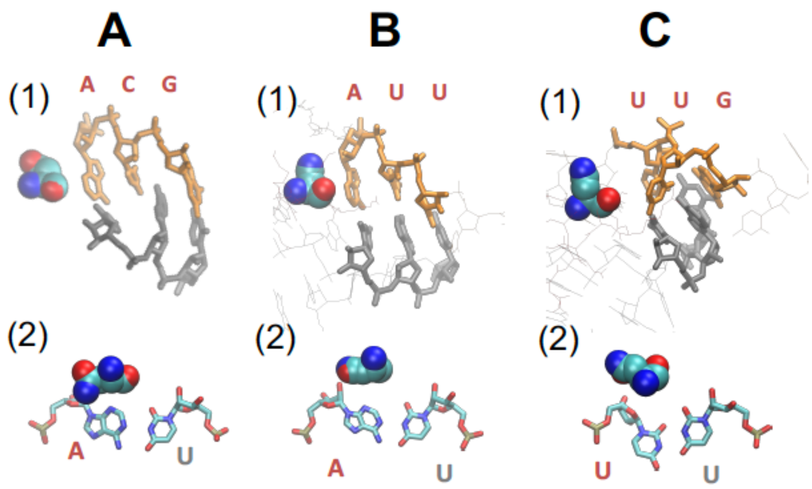

Figure S6. Average structures of the bound state of various start codons. ***Related to Figs. 3 and 5.*** Average structures of the bound state of indicated start codons (orange) are visualized in panel 1 with anticodon bases (gray) and eIF1-N34 (spherical model with color code as in Fig. 5, panels 1. Panel 2, the pair of bases at the first position presented as in Fig. 5, panels 2.

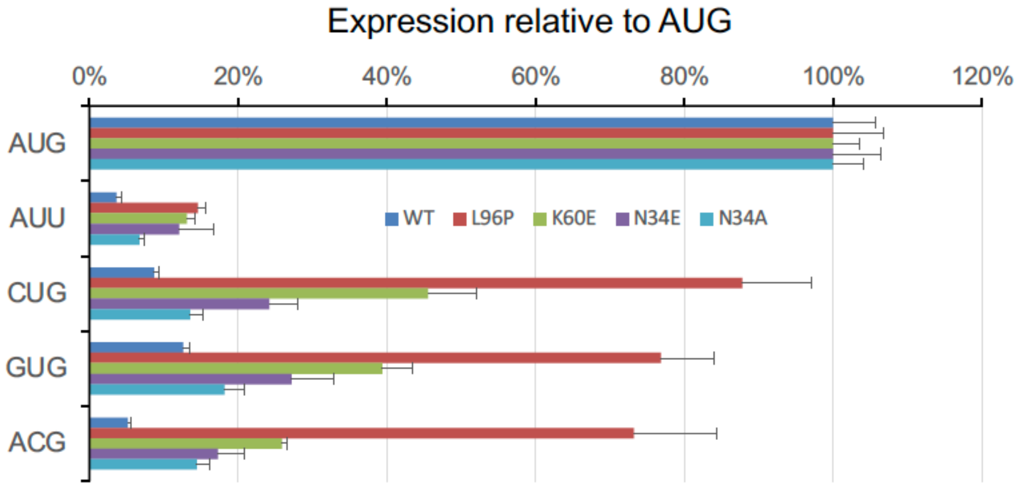

Figure S7. Effect of eIF1 mutations on selection of various start codons in *S. cerevisiae*. *Related to Fig. 7.* Transformants of yeast strains with indicated variants of eIF1 bearing AUG or non-AUG Fluc AUG Rluc dual reporter plasmids were grown in SC-ura at 30° C and subjected to the Dual Glo luciferase assay (Promega), as described in Methods. Obtained Fluc reading was normalized by Rluc expressed from the same plasmid. Values relative to AUG Fluc expression in each strain are presented. Bars indicate SE.
